# Evaluating NfL and NTproBNP as predictive biomarkers of intracranial injuries after mild traumatic brain injury in children presenting to emergency departments

**DOI:** 10.3389/fneur.2025.1518776

**Published:** 2025-01-30

**Authors:** Anne-Cécile Chiollaz, Virginie Pouillard, Michelle Seiler, Céline Habre, Fabrizio Romano, Céline Ritter Schenck, Fabian Spigariol, Christian Korff, Fabienne Maréchal, Verena Wyss, Lyssia Gruaz, Joan Montaner, Jean-Charles Sanchez, Sergio Manzano

**Affiliations:** ^1^Department of Medicine, Faculty of Medicine, University of Geneva, Geneva, Switzerland; ^2^Pediatric Neurology Unit, Woman, Child and Adolescent Department, Geneva University Hospitals, Geneva, Switzerland; ^3^Pediatric Emergency Department, University Children's Hospital Zurich, Zurich, Switzerland; ^4^Division of Radiology, University Hospitals of Geneva, Geneva, Switzerland; ^5^Division of Pediatric Emergency Medicine, Department of Pediatrics, Inselspital, Bern University Hospital, University of Bern, Bern, Switzerland; ^6^Department of Pediatrics, Fribourg Hospital HFR, Fribourg, Switzerland; ^7^Pediatric Emergency Department, Neuchâtel Hospital (RHNE), Neuchatel, Switzerland; ^8^Platform of Pediatric Clinical Research, Woman, Child and Adolescent Department, Geneva University Hospitals, Geneva, Switzerland; ^9^Neurovascular Research Group, Institute of Biomedicine of Seville IBiS/Virgen Macarena University Hospital/CSIC/University of Seville, Seville, Spain; ^10^Pediatric Emergency Department, Geneva University Hospitals and Faculty of Medicine, University of Geneva, Geneva, Switzerland

**Keywords:** blood-biomarkers, mTBI, pediatric, emergency, diagnosis

## Abstract

**Objective:**

Blood-biomarkers have the potential to aid clinicians in pediatric emergency departments (PED) in managing children with mild traumatic brain injury (mTBI) acutely. However, studies focusing on pediatric populations remain limited. We aim to assess the performances of two routinely used biomarkers in other fields: the neurofilament light chain protein (NfL), and the N-terminal prohormone of brain natriuretic peptide (NTproBNP), to safely discharge children without intracranial injuries (ICIs).

**Methods:**

A prospective multicenter cohort study was conducted, enrolling children suffering from mTBI, both with and without imaging during their acute management in the PED. A blood sample was collected within 24 h post-trauma for biomarker analysis. Inclusion criteria followed the PECARN (Pediatric Emergency Care Applied Research Network) guidelines for the diagnosis of mTBI and for ICI on CT as the primary outcome (CT+).

**Results:**

A total of 302 mTBI patients were analyzed comparing children with ICI (18 CT+) versus all the other children without ICI (54 CT− and 230 in-hospital-observation patients without CT). NfL and NTproBNP were increased in the CT+ group and their performances to safely rule-out patient without ICI reached up to 30% specificity with 100% sensitivity. Equivalent performances were observed whether selecting patients with blood collection within 6 h or 24 h post-trauma.

**Conclusion:**

NfL and NTproBNP were described for the first time in children suffering mTBI. Their performances were comparable to well-known biomarkers, such as S100b, GFAP, or HFABP, with the benefit of already being used in routine tests for other diseases. Further large-scale studies are necessary to verify and validate these results.

## 1 Introduction

Every day, pediatric emergency departments (PEDs) are facing admissions of children with mild traumatic brain injury (mTBI), a condition that accounts for approximately 90% of all pediatric TBIs. The worldwide incidence of pediatric TBI ranges between 47 and 280 per 100,000 children (1), with mTBI being defined by a Glasgow Coma Scale (GCS) score of 13–15 (1–3). Despite being classified as mild, mTBI can occasionally lead to intracranial injuries (ICI), such as hemorrhage, requiring surgical intervention (1, 3).

Prompt management of children with mTBI is currently based on clinical decision rules, such as PECARN (Pediatric Emergency Care Applied Research Network) (4). These rules are used to identify children (with GCS 14–15) at very low risk of clinically important traumatic brain injury (ciTBI), who can safely avoid imaging with computed tomography (CT) scans. While clinicians tend to limit unnecessary exposure to ionizing radiation due to the use of CT scans, it is important to notice that children with mTBI still need in-hospital observation for symptoms monitoring. This observation time can last up to 24 h and can be stressful for both children and parents and cost consuming for the health care system. Therefore, improving management for the non-scanned children suffering from mTBI is also needed.

To address this challenge, the study of blood-based biomarkers might provide objective information to guide mTBI patient triage in the PED. Promising evidence supports the integration of biomarkers, particularly S100b, GFAP, or UCHL1 in mTBI management (5). We have recently demonstrated that brain–blood biomarkers (S100b, GFAP, and HFABP) (6), and inflammatory-blood biomarkers (IL6, IL8, and IL10) (7) can safely rule-out mTBI children without ICI with a 100% negative predictive value (NPV). Here, we present two additional promising biomarkers that are already used in clinical practice in other diseases and are therefore readily available for routine blood measurement: the neurofilament light chain protein (NfL), and the N-terminal prohormone of brain natriuretic peptide (NTproBNP).

NfL is a well-established biomarker of axonal injury, released into the cerebrospinal fluid (CSF) and subsequently into the bloodstream following neuronal damage. Its elevation in the blood has been documented across numerous neurologic conditions, including TBI, neurodegenerative diseases, and multiple sclerosis (8, 9). TBI often results in diffuse axonal injury caused by mechanical forces like shearing or stretching. This axonal disruption leads to the release of structural proteins like NfL. Damage to the blood brain barrier (BBB) further allows the leakage of these neuronal proteins into the systemic circulation, making them detectable in peripheral blood. NfL levels tend to rise during the first 2 weeks post-injury and have been so far mainly explored to predict TBI outcome (10). It has been described that NfL blood concentration increases shortly after trauma and correlates with injury severity (11). While NfL has gained attention as a specific and sensitive marker of neuronal damage in adults TBI, its role and clinical utility in pediatric TBI remain less understood.

NTproBNP has been largely studied and described as an important biomarker of cardiac diseases and is routinely used for diagnosing and monitoring heart failure (12). Its precursor BNP is also present in the brain and the CSF (13, 14). After neuronal injuries, the released N-terminal proBNP peptides were found to be elevated in plasma shortly after head injury (15). NT-proBNP levels increase in acute brain injuries, such as stroke and TBI, due to the intricate interaction between the cardiovascular and central nervous systems, known as the brain-heart axis. In severe TBI with intracranial pressure (ICP), CSF-NTproBNP was found elevated at admission and blood-NTproBNP within 12 h post-injury (14). In adults, NTproBNP has already been studied as a potential biomarker of TBI (13) and was found to remain increased during the first 4 days post injury. Its potential application in pediatric TBI has not been systematically explored and further research is essential to elucidate its diagnostic utility and establish age-and condition-specific reference values in the context of TBI.

Our aim was to assess the performances of both NfL and NTproBNP blood concentrations in a prospective multicenter pediatric cohort of patients with mTBI. By accurately identifying children without ICI, these biomarkers could enable safe discharge without the need for CT imaging or observation.

## 2 Methods

### 2.1 Study design and setting

Children were recruited in two prospective multicenter pediatric cohort studies conducted in five PEDs in Switzerland and four PEDs in Spain. Both studies, t-BIOMAP (CCER-ID: 2020–01533) and Biotrabis (BIOTRABIS_FMM-AP171562019) received institutional review board approval and were conducted in accordance with Good Clinical Practice guidelines and provisions of the Declaration of Helsinki. They were registered at www.clinicaltrials.gov: NCT06233851 and NCT04641767.

### 2.2 Patient population

We included all children aged 0 to 16 years who presented to one of the participating PEDs with a mTBI occurring within 24 h prior to presentation.

Written informed consent was obtained from the parents or legal guardians of the children, as well as from the children themselves if they were aged 14 years or older.

Inclusion criteria were defined as: (1) a GCS score of 14; or (2) a GCS score of 15 with at least one of the following symptoms: loss of consciousness (LOC) for <30 min, post traumatic amnesia (PTA) lasting <24 h, persistent headaches, irritability, three or more episodes of vomiting, confusion, vertigo or dizziness, post-traumatic seizure, or transient neurological abnormality; or (3) GCS score 15 with signs for basal skull fracture; or (4) GCS score 15 with high-energy trauma (traffic accident or a fall of >0.9 m in children <2 years old, or > 1.5 m in children ≥2 years old). Exclusion criteria included: participation in another clinical study with pharmacologic treatment, alcohol consumption or use of psychoactive substances, a history of recent TBI (within the last month), recent history of epileptic seizures (within the last month), Down syndrome (because the gene encoding for the S100b protein is located in the Down Syndrome Critical Region (DSCR) of the 21 chromosome), acute encephalopathy, encephalitis, meningitis, or refusal to give their consent.

A cohort of healthy children was also included in the study. Eligibility criteria required participants to be 16 years old or younger, have a scheduled blood draw in the ambulatory care unit, and have no history of TBI. The exclusion criteria matched those established for the TBI group.

### 2.3 Intervention and blood biomarker analysis

After informed consent was obtained, blood samples were drawn as soon as possible, but no later than 24 h after the trauma. Serum samples were obtained by centrifugation and stored at −80°C. NTproBNP and NfL concentrations were measured using enzyme-linked immunoassay (ELISA): Rplex Human NTproBNP (F214I) and Rplex Human Neurofilament L (F217X) Antibody Sets (Meso Scale Diagnostics, Rockville, MD, USA). Lower limits of detection (LLoD) were 5.5 pg/mL with a calibration range of 12.21–50,000 pg/mL for NfL, and 0.30 pg/mL with a calibration range of 0.12–500 pg/mL for NTproBNP. Lower limits of quantification (LLoQ) were < 12.21 pg/mL for NfL and 0.49 pg/mL for NTproBNP, respectively. All kits were used according to the manufacturers’ instructions. Duplicate control serum samples were measured on each plate, with intra-and inter-plate coefficients of variation (CVs) below 20%.

The study did not interfere with any medical decision-making.

### 2.4 Data collection

Study data were collected and managed using REDCap electronic data capture tools hosted at Hôpitaux Universitaires de Genève (HUG) (16, 17). Clinical records included: sex, age, GCS score, causes of injury, history of coagulation disorders with medication intake prior to trauma, TBI-associated symptoms, simple skull fractures, presence of extracranial injuries (ECIs) such as other body fractures or organs injuries, time between trauma and blood sampling, physician decision regarding patient management (observation without CT scan or undergoing CT scan), effective time in observation in the PED for symptom monitoring, neurosurgery and intubation if needed, and results of imaging.

### 2.5 Outcome measures

The primary outcome was the presence of ICI on CT. PECARN criteria (4) were used to define ICI on CT images. These criteria included: intracranial hemorrhage or contusion, cerebral edema, traumatic infarction, diffuse axonal injury, shearing injury, sigmoid sinus thrombosis, midline shift of intracranial contents or signs of brain herniation, diastasis of the skull, pneumocephalus or skull fracture depressed by at least the width of the skull table. All CT scans were analyzed by the same pediatric radiologist (CH) who was blinded to clinical presentation and biomarker result. The presence of any of the findings listed above was defined as CT+, and the absence of them was defined as CT−. For non-scanned patients, who might have experienced a decline within 48 h following their trauma, a return visit to the PED would have been documented, and this information was available in the clinical report form.

The diagnostic values of the blood biomarkers were evaluated to determine their ability to rule-out children without ICI, by comparing CT+ patients versus both CT− and in-hospital observation patients.

### 2.6 Statistical analysis

Statistical analysis was performed using R^1^ in RStudio^2^. Biomarker concentrations were normalized using their medians as correction factors. Patients were dichotomized into two groups: (1) CT− and in-hospital-observation without CT, (2) CT+ patients. Differences between groups were established using the nonparametric Mann–Whitney *U* test, given that the Kolmogorov–Smirnov test revealed that all protein levels were non-normally distributed (*p* < 0.05). Chi-squared test was used for statistical analyses of the clinical data. Statistical significance was inferred at *p* < 0.05. The levels of biomarkers are presented using box-and dot-plots with a log10 Y-scale. Biomarker’s ability for classifying patients according to their group was evaluated using receiver operating characteristic (ROC) curves with the pROC package in R. For each biomarker, the optimal performance was identified by maximizing specificity while maintaining 100% sensitivity, aiming to safely identify the maximum of patients without ICI. The main analysis included all patients with blood sampling within 24 h post-trauma, with sub-analysis performed on patients with blood sampling within 6 h post-trauma. Equivalent analyses were also conducted including only CT-scanned patients (CT− versus CT+). The correlation between blood-protein concentrations and patient age were evaluated in the healthy population, by Spearman correlation coefficient and its *p*-value.

## 3 Results

A total of 302 mTBI children (mainly Caucasian) were included between October 2020 and February 2023, with blood samples collected within 24 h of trauma. Among these, 18 (6%) patients were positive on CT-scans (CT+), 54 patients (18%) were negative (CT−), and the remaining 230 patients (76%) were kept for observation without imaging (in-hospital-observation) (Table 1).

**Table 1:** Clinical parameters and biomarkers expression in mTBI patients with and without CT scan (within 24 h).

*N*=302 mTBI patients	CT- or Observation (*N*=284) *94%*	CT+ (*N*=18)*6%*	*p*-value
**Age (yo)**			
Mean (SD)	8.53 (4.50)	8.04 (4.85)	0.72
Median [Min, Max]	8.85 [0.100, 16.0]	8.20 [0.110, 15.0]	
**Sex, n (%)**			
Boys	162 (57.0%)	13 (72.2%)	0.308
Girls	122 (43.0%)	5 (27.8%)	
**Severity of injury, n(%)**			
GCS 14	29 (10.2%)	4 (22.2%)	0.232
GCS 15	255 (89.8%)	14 (77.8%)	
**Associated symptoms, when GCS 15, n (%)**			
Loss of consciousness	56 (19.7%)	1 (5.6%)	0.319
Post-traumatic amnesia	85 (29.9%)	1 (5.6%)	0.076
Persistent headaches	84 (29.6%)	7 (38.9%)	0.317
More than three episodes of vomiting	53 (18.7%)	6 (33.3%)	0.111
Vertigo	23 (8.1%)	1 (5.6%)	1
Confusion	37 (13.0%)	1 (5.6%)	0.691
Convulsion	5 (1.8%)	0 (0%)	1
**Extracranial injuries (ECI), n (%)**			
mTBI + others body lesions or fractures	36 (12.7%)	4 (22.2%)	0.229
**Simple Skull fracture (on CT), n (%)**			
yes	12 (4.2%)	15 (83.3%)	**<0.001**
**time lap TBI-blood sampling (hour)**			
Mean (SD)	6.24 (4.59)	9.91 (7.36)	**0.039**
Median [Min, Max]	5.00 [1.00, 24.0]	8.00 [2.00, 24.0]	
**NfL (pg/ml)**			
Mean (SD)	23.3 (19.0)	90.6 (145)	**<0.001**
Median [Min, Max]	17.7 [0.119, 127]	41.4 [13.1, 650]	
Missing	1 (0.4%)	0 (0%)	
**NTproBNP (pg/ml)**			
Mean (SD)	88.0 (80.2)	175 (218)	0.079
Median [Min, Max]	65.9 [1.99, 605]	63.3 [30.7, 915]	
Missing	4 (1.4%)	0 (0%)	

CT- (*n* = 54) and in-hospital-observation (*n* = 230). Bold values represents significant *p* value < 0.05.

The ages of patients ranged from 1 month to 16 years in the CT− and in-hospital-observation groups, and from 9 months to 15 years in the CT+ group. The mean age was 8 years across both groups. The majority of patients had a GCS of 15 with associated symptoms. The most prevalent associated symptoms were PTA, persistent headaches, LOC, and more than three episodes of vomiting, without significant differences observed between the CT− and in-hospital observation groups compared to the CT+ group (Table 1). The presence of simple skull fractures (i.e., linear, non-displaced) seen on CT was significantly higher in the CT+ patient group (*p* < 0.001). No other significant differences in clinical parameters were observed by comparing the CT− and in-hospital-observation patients’ group to the CT+ patients’ group.

The median time from head trauma to blood sampling was 8 h for CT+ patients and 5 h for the other mTBI patients, with this difference being significant (*p* = 0.039).

**Figure 1:**
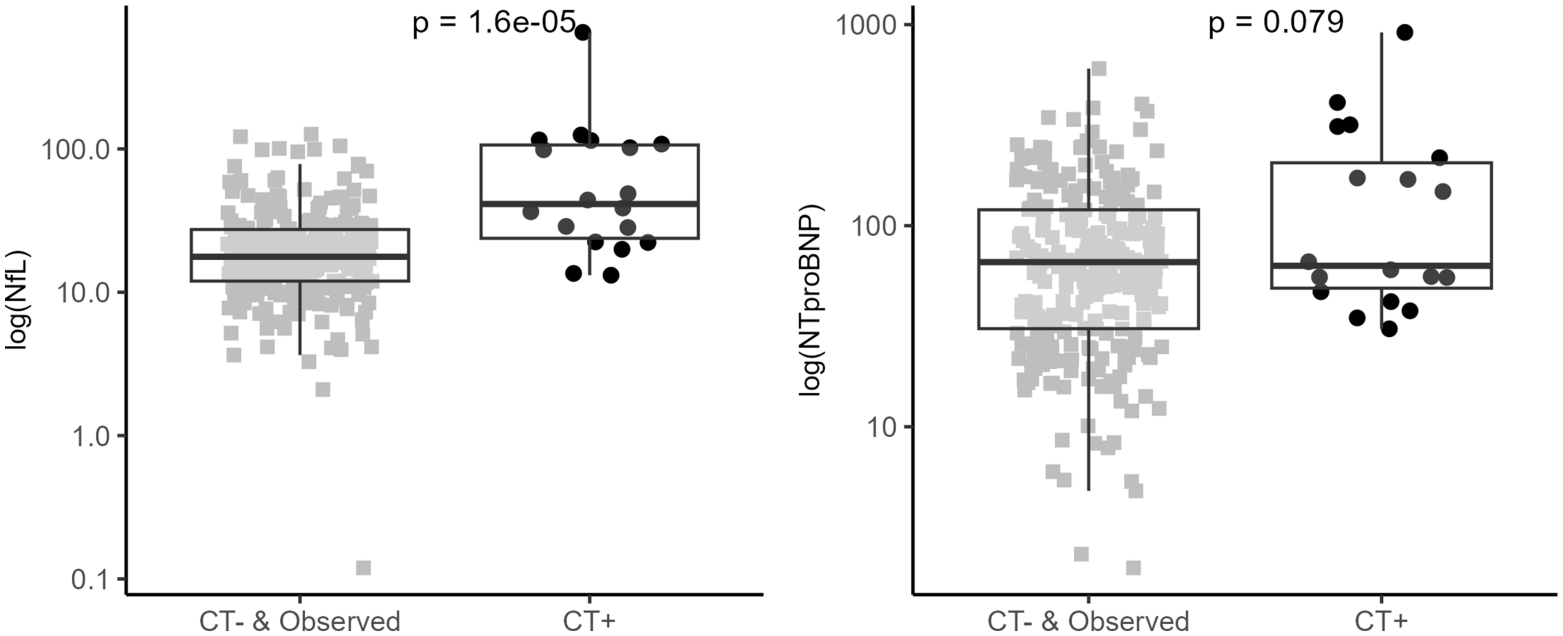
NfL and NTproBNP serum concentration in mTBI patients (within 24 h). Biomarkers expression within CT− or in-hospital-observation patients *(grey square)* and CT+ *(black round)* mTBI patients. Box plots represent median and IQR for compared groups; dot plots represent for each patient log scaled biomarker’s concentration. The analysis was carried out using a Mann–Whitney *U* test (shown *p*-value). Positive CT is based on PECARN criteria.

NfL and NTproBNP means with SD and medians with minimum and maximum are reported in Table 1. Blood concentrations of both biomarkers were increased in CT+ patients compared to CT− and in-hospital-observation patients (Figure 1 and Table 1). This increase in NfL concentration was significant (*p* < 0.001), while the increase in NTproBNP concentration was not (*p* = 0.079).

**Figure 2:**
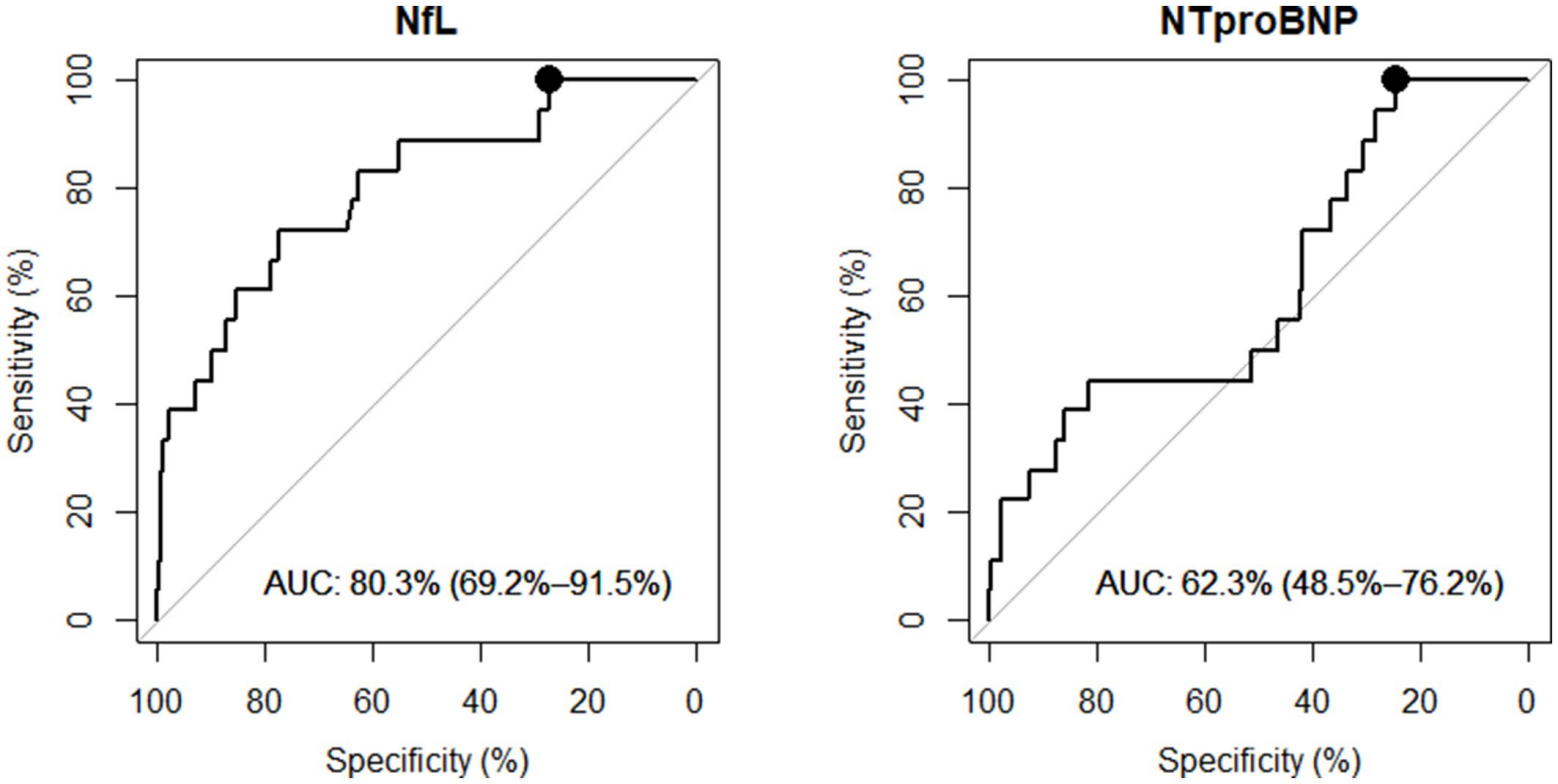
NfL and NTproBNP diagnostic performances to classify mTBI patients (within 24 h). Receiver Operating Characteristic (ROC) Curves comparing CT− or in-hospital-observation patients versus CT+ mTBI patients. AUC, Area Under the Curve with 95% confidence interval. Performances were investigated at 100% sensitivity and corresponding highest specificity (*black round* on ROC curve).

The diagnostic performance of the biomarkers is presented with ROC curves in Figure 2. To accurately identify CT− and in-hospital-observation patients, NfL yielded a specificity of 27% [95% IC: 22–32%] and NTproBNP demonstrated a specificity of 25% [95% IC: 18–30], both with 100% sensitivity (Table 2).

**Table 2:** NfL and NTproBNP best performances in discriminating mTBI with ICI.

Biomarkers	Time lap TBI-blood sampling-number of patients	Sensitivity (%) (95% CI)	Specificity (%) (95% CI)	AUC (95% CI)	Threshold (pg/ml)
**NfL**	≤ 24 hours *n = 302*	100 (100 – 100)	**27.21** (22.19 – 32.32)	80.3 (69.2 – 91.5)	13.13
	≤ 6 hours *n = 222*	100 (100 – 100)	**31.16** (25.07 – 37.25)	67.7 (47.8 – 87.5)	13.13
**NTproBNP**	≤ 24 hours *n = 302*	100 (100 – 100)	**24.64** (18.78 – 29.50)	62.3 (48.5 – 76.2)	30.68
	≤ 6 hours *n = 222*	100 (100 – 100)	**25.59** (19.85 – 31.33)	55.4 (34.2 – 76.5)	30.68

Within 24 hours post-trauma: CT− and in-hospital-observation (*n* = 284); CT+ (*n* = 18) - within 6 hours post-trauma: CT− and in-hospital-observation (*n* = 215); CT+ (*n* = 7).

In the sub-analysis focusing on patients with blood sampling within 6 h post-trauma, a total of 222 children were included. Out of them, 179 patients (80%) were kept for observation, and 43 patients (20%) underwent CT scanning, with 7 patients (3%) having a CT+ result. In this subgroup, NfL exhibited a specificity of 31% [95% IC: 25–37%], and NTproBNP a specificity of 26% [95% IC: 20–31%], both with 100% sensitivity to discriminate CT− and in-hospital-observation patients versus CT+ patients. These performances are summarized in Table 2. Notably, NfL and NTproBNP specificities remained stable around 30% regardless of whether the sampling was done within 6 or 24 h, with a negative predictive value (NPV) of 100%.

Additionally, we investigated the performance of the biomarkers exclusively in the CT-scanned subgroup. NfL demonstrated a specificity of 24% [95% IC: 14–34%] and NTproBNP showed a specificity of 35% [95% IC: 24–46%] for identifying CT− patients, both with 100% sensitivity (Table 3). For patients with blood sampling performed within 6 h post-trauma, there were 35 CT− patients and 7 CT+ patients. NfL exhibited a specificity of 31% [95% IC: 17–44%] and NTproBNP a specificity of 41% [95% IC: 27–56%] (Table 3).

**Table 3:** NfL and NTproBNP best performances in discriminating mTBI with ICI only within CT-scanned patients.

Biomarkers	Time lap TBI-blood sampling-number of patients	Sensitivity (%) (95% CI)	Specificity (%)(95% CI)	AUC (95% CI)	Threshold (pg/ml)
**NfL**	≤ 24 hours *n = 72*	100 (100 – 100)	**24.07** (14.20-33.94)	74.5 (61.1-87.9)	13.12
	≤ 6 hours *n = 42*	100 (100 – 100)	**30.56** (16.79-44.33)	58.7 (38.1-79.3)	13.12
**NTproBNP**	≤ 24 hours *n = 72*	100 (100 – 100)	**34.62** (23.63-45.61)	64.4 (50.2-78.5)	29.99
	≤ 6 hours *n = 42*	100 (100 – 100)	**41.18** (26.47-55.89)	62.6 (42.0-83.2)	27.95

Within 24 hours post-trauma: CT− (*n* = 54); CT+ (*n* = 18) - within 6 hours post-trauma: CT− (*n* = 35); CT+ (*n* = 7).

A slight negative age-correlation was found in the healthy cohort of 99 children, for both NfL and NTproBNP (Spearman *r* = −0.22, *p* = 0.028 for NfL and Spearman *r* = −0.06, *p* = 0.520 for NTproBNP).

## 4 Discussion

This study is the first to evaluate the biomarkers NfL and NTproBNP in a pediatric cohort of mTBI patients. Notably, it includes non-scanned patients, who represent nearly 80% of the mTBI patients managed in the PEDs. This is also the first study to focus on children staying in observation in the PED, a group of whom clinicians currently lack tools to facilitate earlier discharge.

NfL and NTproBNP could identify up to 30% of mTBI patients without ICI, while successfully detecting all CT+ patients (100% NPV). This indicates that nearly one third of mTBI children could be safely discharged earlier from the PED and avoiding unnecessary CT scans. In this same cohort, we previously studied the diagnostic performance of the well-known S100b protein, confirming its 34% specificity with 100% NPV in scanned patients (6, 18, 19). While S100b is effective when measured early after trauma [within 3 to 6 h (20, 21)], our results demonstrate that NfL and NTproBNP remained stable within 6 or 24 h. This stability makes them viable options for use later after trauma onset. Both biomarkers are known to rise after adult TBI and remain elevated for several days (13, 22). Here we demonstrated that NfL and NTproBNP blood levels also increased after pediatric TBI.

In our cohort, there was a significant difference in the timing of blood collection between the CT+ and CT− groups within the ≤24 h selection; however, this difference was not observed in the ≤6 h selection (Medians: 3 h for CT+ patients and 4 h for other mTBI patients, *p* = 0.097). The presence of intracranial injuries in CT+ patients likely necessitated rapid management to address their critical needs, resulting in delayed blood drawing for the research study.

Another advantage of NfL and NTproBNP compared to more established biomarkers such as S100b, GFAP, or HFABP, is their availability in routine laboratories.

However, despite their comparable performance, we are aware that this is not sufficient for clinical use. To increase specificity, these blood-biomarkers could be combined into a panel of biomarkers, potentially increasing their diagnostic performance by capturing different ICI signatures after mTBI. Such panels might also include clinical variables such as age, gender, or even GCS. In adult mTBI, panels such as GFAP + UCHL1 (23) or GFAP + HFABP (24) have already been published.

Our study differs from previous studies in adult mTBI, because we included non-scanned patients, and looked at biomarker’s specificity with 100% sensitivity to safely rule-out a maximum of patient. We therefore aimed to reduce the length of stay in observation at the PED for mTBI patients without ICI, which might have a double impact: improving the well-being of children and their families while reducing PED overcrowding.

The major limitation of this study is the small number of patients in the CT+ group, which does not allow further stratified analyses, nor consider ciTBI as the primary outcome, as it is proposed in the PECARN studies. ciTBI refers to any of the following descriptions: death, neurosurgical intervention, intubation of more than 24 h, or hospital admission of 2 nights or more for the TBI in association with ICI on CT. Since ciTBI occurs in less than 1% of the mTBI cases, larger multicenter cohorts of pediatric mTBI patients are required to increase the size of the ciTBI+ group and to be able to assess this more clinically meaningful outcome. Nevertheless, this study provides a first step toward the achievement to better manage mTBI children. To further align with the PECARN rules, stratifying results by age group (<2 years and ≥ 2 years) would have been required but was impaired by the sample size. Age-stratification is known to be needed to establish appropriate cutoff for different age groups, as already demonstrated for S100b in newborns (25). This will be particularly important for both NTproBNP and NfL as they have shown to vary significantly with age in healthy children, as noted here and in the CALIPER study (26, 27). This large pediatric study aimed at deciphering and establishing pediatric serum biomarkers concentrations in healthy pediatric control and underscores the importance of using pediatric-specific reference values when utilizing biomarkers (27).

Another limitation is the reliance on research-based immunoassays. To ensure verification and validation, additional studies may need to be conducted directly on validated analyzers within hospital laboratory medicine.

## 5 Conclusion

This study describes the impact of blood-based biomarkers to improve pediatric mTBI management. NfL and NTproBNP achieved equivalent performances with well-known brain trauma biomarkers to safely rule-out one third of pediatric mTBI patients without ICI, and with the benefit of being available in routine testing. However, to achieve integration in clinical practice, efforts should be directed to combine different biomarkers in order to increase specificity in larger scale studies.

## Data Availability

The raw data supporting the conclusions of this article will be made available by the authors, without undue reservation.

## Footnotes

^1^http://www.rproject.org, version 4.3.0

^2^http://www.rstudio.com, version 2023.06.0
